# How Does Woodland Use Affect the Multifunctionality of Soil Ecosystems?

**DOI:** 10.3390/microorganisms14030685

**Published:** 2026-03-18

**Authors:** Jing Li, Jun Yao, Nan He, Deliang Zhang, Jing Zhang, Xingyuan Ma

**Affiliations:** 1 College of Forestry, Southwest Forestry University, Kunming 650224, China; henan-xw@swfu.edu.cn (N.H.); 18257359788@163.com (D.Z.); 19903404847@163.com (J.Z.); m15911918153@163.com (X.M.); 2 College of Agriculture and Forestry Science and Technology, Yuxi Vocational College of Agriculture, Yuxi 653100, China; eyaojun@hotmail.com

**Keywords:** woodland use intensity, plant diversity, soil microbial communities, soil ecosystem multifunctionality

## Abstract

Humans have made tremendous efforts to explore how biodiversity changes affect ecosystem processes and human well-being. It has been found that, in addition to biodiversity, various drivers of global change also play significant roles in ecosystem functioning. Land use is a key driver of global change, yet research on land use intensity has predominantly focused on agricultural and grassland ecosystems. There remains limited understanding of how land use intensity alters the relationships among biodiversity, ecosystem functions, and multifunctionality, particularly in forest ecosystems. This paper reviews recent advances in research on soil ecosystem multifunctionality, covering the effects of woodland use intensity, above- and belowground biodiversity, microbial diversity, and biotic interactions, as well as abiotic drivers. Through a comprehensive analysis of the integrated impacts of biodiversity, biotic interactions, and abiotic factors on soil ecosystem multifunctionality, the necessity of enhancing microbial research and its application in ecosystems is emphasized, providing a theoretical basis for forest management.

## 1. Introduction

Humans continuously modify the Earth’s surface environment to obtain resources, resulting in diverse land use patterns [1]. Land use refers to human activities directly related to the land, that is, activities that utilize its resources or have an impact on it [2]. Changes in land use intensity (LUI) (i.e., an increase in land use intensity), such as an increase in agriculture, mineral extraction, frequent mowing of grass, or an increase in grazing intensity, often pose a significant threat to biodiversity [3,4]. Changes in LUI have resulted in the loss of plant species diversity and a decline in soil functions, such as soil nitrogen and moisture retention [5]. LUI is one of the principal drivers of global biodiversity loss. It strongly alters terrestrial ecosystems worldwide [6,7] and compromises the multiple ecosystem functions they provide—such as soil nutrient cycling and water retention [8]—with consequent impacts on human well-being [8,9]. The decline in biodiversity impacts both aboveground and belowground plant and microbial communities, thereby influencing soil ecosystem multifunctionality [8,9].

“Ecosystem multifunctionality (EMF)” was first introduced as a term in 2004 [10]. In 2007, Hector and Bagchi [11] defined ecosystem multifunctionality as the ability of ecosystems to simultaneously provide and maintain multiple ecosystem functions and ecosystem services. Because trade-offs exist among ecosystem function, managing ecosystems from the perspective of a single ecosystem function can compromise the provision or maintenance of other functions [12]. In recent years, researchers have attempted to represent the overall functioning of ecosystems with a single value [10,11], aiming to clarify ecosystems’ capacity and performance in simultaneously providing and maintaining multiple ecosystem functions [11,13,14,15]. This shift has moved related research from focusing on drivers of single ecosystem functions [13,16] to understanding the drivers of multiple ecosystem functions [17], thereby advancing the field into a significant new stage. Studying ecosystem multifunctionality can elucidate how factors such as biodiversity, environmental variables, and land use changes simultaneously affect multiple ecosystem function. Since then, Ecosystem multifunctionality has been widely adopted. Manning et al. [15] defined multifunctionality at two hierarchical levels: (1) Ecosystem function multifunctionality, which involves a series of biological, geophysical, and chemical processes occurring within ecosystems and is most closely related to the fundamental research on drivers of ecosystem functions, and (2) Ecosystem service multifunctionality, which refers to the simultaneous provision of multiple ecosystem services that meet human needs and is most relevant to stakeholders with explicit management objectives. This framework distinguishes between the ecosystem function multifunctionality and the ecosystem service multifunctionality, facilitating their differentiation and quantification. EMF provides a novel and integrated perspective for ecosystem management. While domestic research on forest EMF has been increasing [18], studies focusing on the impact of different land use practices on ecosystem multifunctionality remain relatively scarce [19]. This gap hinders the full realization of ecosystem functions following forest conversion [20]. Consequently, investigating the multifunctionality of forest ecosystems is of paramount importance for a systematic and comprehensive understanding of ecosystem functions.

LUI can significantly and directly affect soil ecosystem multifunctionality, as well as indirectly influence soil ecosystem multifunctionality through changes in biodiversity and structural functions [17]. The increase in LUI can reduce soil ecosystem multifunctionality through the interaction between plant diversity and soil bacterial diversity [21]. Increasing LUI alters key components that are crucial for ecosystem function and changes the synergy between biodiversity and ecosystem function [22]. LUI affects biodiversity and ecosystem functions to varying degrees, while also influencing the relationship between them and the regulatory factors. However, research on LUI has primarily focused on agricultural and grassland ecosystems. Little is known about how forest LUI changes the relationship between biodiversity, ecosystem function, and multifunctionality, especially in forest ecosystems. Therefore, this study clarifies the relationship between woodland use intensity and soil ecosystem multifunctionality by reviewing the impacts of woodland use on aboveground plants, underground microorganisms, and the effects of both plants and microorganisms on ecosystem functions and multifunctionality.

The effects of land use change on biodiversity and ecosystem functions largely depend on the type, timing, frequency, and intensity of land use disturbances [7]. Changes in woodland use constitute an important component of global change, particularly land use change [23], and woodland use intensity (WUI) is also a critical factor influencing ecosystem structure and function [21,24]. WUI can alter vegetation cover and cause soil disturbance and erosion, thereby affecting the composition of soil microbial communities [19]. Changes in WUI inevitably influence both biotic and abiotic factors in forest ecosystems, making it essential to understand the effects of biotic and abiotic factors on ecosystem function and multifunctionality. In addition to the direct biological effects of plant and microbial diversity on soil ecosystem processes (through resource inputs), abiotic conditions such as temperature, humidity, soil moisture content, and soil pH play a crucial role in shaping soil microbial communities and their functions (e.g., decomposition rates and dynamics), which, in turn, affect biodiversity and ecosystem function [25]. Biotic and abiotic factors can alter the structure and function of organisms by affecting their composition, growth, and productivity, while changes in climate or human management practices also have significant impacts on ecosystems [26]. Seasonal variations, primarily associated with temperature and precipitation, result in hydrothermal differences that constitute key environmental drivers influencing biodiversity and ecosystem function [27], with notable effects on community structure and diversity [28,29]. Changes in species diversity and composition are driven by various environmental shifts, including land use, nutrient availability and cycling, atmospheric composition, climate, introduction of non-native species, and human overexploitation, ultimately affecting ecosystem function and multifunctionality [28,29,30,31,32]. van der Plas et al. [33] demonstrated that relying solely on biodiversity does not adequately predict ecosystem function, highlighting the importance of incorporating abiotic factors in studies of ecosystem processes. How does woodland use affect ecosystem function and multifunctionality?

## 2. Effect of Woodland Use Intensity on Soil Microbial Diversity, Community Composition, and Functional Guilds

Woodland use changes affect microbial composition and abundance by altering soil properties and removing protective vegetation [19]. Woodland use conversion in forested areas can impact plant diversity, community structure, and soil properties [34]. The conversion from monsoon evergreen broad-leaved forests to plantations disturbs stable ecosystems, thereby enriching microbial species in the soil [24,35]. Due to interactions between aboveground plants and belowground microorganisms, changes in WUI significantly affect soil microbial diversity and community composition [19]. Plant community composition and diversity can directly influence the composition and diversity of soil microbial communities by altering root exudates, plant litter, and ecological niches available to microorganisms [36,37,38]. Belay-Tedla et al. [39] found that a decrease in soil total nitrogen may lead to increased root exudation and litter input, which in turn enhances soil microbial diversity. Vegetation composition and diversity can also indirectly affect microbial communities by modifying soil properties [37]. Microorganisms play a crucial role in the phosphorus cycle. Li et al. [19] demonstrated that soil available phosphorus positively influences soil bacterial diversity, while soil total phosphorus positively affects fungal diversity [40]. Therefore, plant species richness and soil properties are significant in regulating the diversity of fungal and bacterial communities.

The composition and distribution of soil fungal communities are influenced by aboveground vegetation and belowground soil properties [41]. Studies have found that WUI can affect the increase in soil fungal diversity and the decrease in community similarity through soil properties (C/N) and aboveground plant species richness; and significantly affects the increase in soil bacterial diversity and the increase in community similarity through phosphorus and potassium elements and aboveground plant species richness [42]. Basidiomycota fungi can decompose carbon and wood with high levels of complex carbon compounds through the biosynthesis of several enzymes and participate in carbon cycling in various forest environments [43]. Changes in soil fungal community structure are associated with significant reductions in plant species richness and soil carbon and nitrogen levels [44], highlighting the important role of the quantity and quality of plants and soil nutrients in studying fungal community composition and structure [43]. Many tree species in global forest habitats rely on symbiotic ectomycorrhizal fungi to meet their nutritional needs [45]. These fungi serve as a crucial pathway for carbon input into the soil and constitute a significant component of forest soil carbon fluxes [41] (Table 1). Therefore, fungi are recognized as key drivers of forest ecosystem processes and essential microorganisms in forest nutrient cycling [45]. In natural forests and *Pinus kesiya* forests, ectomycorrhizal fungi can form symbiotic relationships with plants such as Fagaceae, Lauraceae, and Pinaceae, leading to a relatively high abundance of ectomycorrhizal fungi in these forest types. After woodland conversion, the number of tree species capable of symbiosis with ectomycorrhizal fungi decreases, resulting in a reduced relative abundance of ectomycorrhizal fungi. Human-induced disturbances can also reduce the relative abundance and richness of ectomycorrhizal fungi and lower soil extracellular enzyme activity [46]. Changes in specific fungal communities may affect multiple ecosystem functions, including carbon and other nutrient cycling processes [47]. The transformation from ectomycorrhizal fungi to saprophytic fungi will alter the soil carbon and nitrogen cycles. For example, enhanced soil nutrient decomposition can reduce soil carbon and sequester soil nitrogen within saprotrophic microbial biomass [45]. Although saprotrophic fungi do not dominate in relative abundance during woodland use processes, they are ecologically significant. The main reason is that saprophytic fungi are dominant in the litter layer and share overlapping ecological niches with ectomycorrhizal fungi [48]. Therefore, ectomycorrhizal fungi can suppress the activity of saprotrophic fungi in regulating litter decomposition and reduce their relative abundance [49].

Soil bacteria dominate the composition of soil microorganisms (Table 1). Acidobacteria and Proteobacteria play significant roles in soil nutrient cycling (C, N, and P) and the decomposition of organic matter [50]. Phosphorus is one of the essential nutrients for plant growth and development, participating in plant cell mitosis as well as the synthesis of plant matter and energy [51]. Soil phosphorus content is closely linked to the presence of Acidobacteria and Proteobacteria [52]. These bacterial groups facilitate plant uptake of phosphorus, indicating that soil phosphorus content significantly influences both plants and soil microorganisms. Most bacterial functional groups are affected by plant species richness and soil properties [19]. Soil phosphorus and potassium content primarily influence chemoheterotrophic, aerobic chemoheterotrophic, cellulolytic, and ureolytic bacterial functional guilds, whereas the soil C:N ratio mainly affects animal parasitic symbiotic and nitrification bacterial functional guilds. Soil bacterial communities play a crucial role in biogeochemical cycles [53]. They mediate key nitrogen transformation processes such as biological nitrogen fixation, ammonification, nitrification, and denitrification [54]. Land degradation resulting from changes in woodland use leads to alterations in soil properties, which, in turn, affect the functional composition of soil bacteria. Changes in microbial functional composition can further influence soil carbon and nitrogen transformation processes. The impact of soil bacterial diversity on plant productivity can be either positive or negative, depending on environmental conditions, spatial and temporal scales, and soil fertility [55]. Negative effects may be attributed to competition between microorganisms and plants for limited nutrients, particularly in nitrogen- and phosphorus-limited ecosystems. Hu et al. [56] found that an increase in soil bacterial diversity may reduce the dominance of specific prokaryotic groups with specialized functions, thereby diminishing their contribution to ecosystem functions (e.g., nutrient cycling mediated by bacteria involved in ammonia oxidation) [57]. Alternatively, it may decrease plant-available resources through nutrient loss [58], thereby slowing nutrient supply and resource reuse in the soil [59]. This may further reduce their interactions with other microorganisms or intensify competition with other microbes and plants for nutrients, ultimately leading to negative effects on plant productivity and related soil functions [60].

**Table 1:** The relative roles of fungi and bacteria in maintaining soil multifunctionality.

Function	Fungi	Bacteria
Carbon storage and sequestration	By forming stable microbial residue carbon, slowing down the decomposition of organic matter, and promoting carbon sequestration [61].	Responsible for rapid carbon mineralization and maintaining energy flow [5].
Nitrogen cycle	By competing for nitrogen sources, it regulates the rate of nitrogen transformation in bacteria [62].	Dominates key processes such as nitrogen fixation, nitrification, and denitrification [63].
Phosphorus cycle	Secretes phosphatase to dissolve organophosphorus and improve phosphorus availability [64].	Participates in phosphorus mineralization and maintains phosphorus balance [64].
Water retention and soil structure	Fungal hyphae (such as ectomycorrhiza) can form soil aggregates, enhancing the water retention capacity [65].	It participates in the formation of aggregates by secreting substances such as extracellular polysaccharides [66].
Enzymatic activity	Produces specific lignin enzymes and phosphatases [67].	Produces a wide range of extracellular enzymes (such as β-glucosidase, aminoglycoside enzymes) [68].
Network complexity	Mycorrhizal networks are the crucial framework of soil microbial networks, and they exhibit high complexity, which leads to their strong versatility [69].	Bacteria provide active metabolic nodes in the network [70].

Soil microbes form a complex, interconnected community that both shapes and is shaped by soil properties. They can affect aboveground ecosystems by promoting plant nutrition, altering soil structure, and modifying soil fertility [71]. Consequently, soil microorganisms respond to management regimes and environmental shifts far more rapidly than the soil properties. Alterations in soil microbial composition serve as a foundation for evaluating soil functional integrity [72]. Soil microbial diversity determines soil fertility, productivity, and ecological stability [73]. The key factors influencing soil microbial abundance include the environment, soil nutrient status, soil pH, soil texture, the rhizosphere, and host plant [71]. Plant species, plant community diversity, and microbial interactions significantly shape the structure and composition of soil microbial communities. Since low plant diversity communities often result from human activities, anthropogenic disturbances reduce forest cover, alter forest soil microbial habitats, and—coupled with soil nutrients—affect microbial community composition and diversity [73]. Soil microorganisms are closely linked to biogeochemical cycles [74,75] and underpin soil ecological processes and ecosystem functions. Soil microbial diversity can modulate the effects of anthropogenic disturbances and environmental changes on soil ecosystems [57]. They are regulated by abiotic factors such as soil pH, texture, nutrient availability, and moisture [76]. Soil pH [30], soil water content [49], and other soil factors [29] are also primary drivers of soil ecosystem multifunctionality. Overall, forest land use and management can alter the availability and turnover of soil nutrients, thereby modifying soil functions [76].

## 3. Woodland Use Intensity Affects Soil Ecosystem Multifunctionality Through Biodiversity and Network Complexity

Increased plant diversity provides more resources such as litter and root exudates [77], resulting in the accumulation of soil nutrients, improved soil water retention, and enhanced soil nutrient cycling [21]. Plant communities with higher diversity are better able to capture resources and convert them into new biomass [78], as co-occurring species with contrasting trait values enhance overall resource acquisition and utilization through niche complementarity [79]. A larger proportion of carbon in plants is allocated belowground to roots and associated fungi, thereby influencing organic matter turnover, carbon sequestration, and nutrient dynamics belowground [78]. Thus, plant diversity plays a significant role in forest soil ecosystem multifunctionality.

Soil microorganisms play a crucial role in nutrient cycling and soil structure formation in terrestrial ecosystems, promoting the delivery of multiple ecosystem functions [80]. Soil fungi are indispensable in the decomposition of complex carbon compounds within ecosystems [49]; for instance, saprophytic fungi can break down recalcitrant organic matter, whereas bacteria are associated with the turnover of readily degradable substrates [81]. Diverse resource inputs from plant communities stimulate soil microbial activity, thereby enhancing the production of certain extracellular enzymes and the release of available soil nutrients, which increases soil ecosystem multifunctionality [78]. Conversely, nutrients released from organic matter decomposition become available for aboveground plants, thus aiding in the restoration of plant communities [81]. Li et al. [19] found that forest land use intensity can significantly affect soil ecosystem multifunctionality through microbial symbiotic networks (fungal–fungal, bacterial–bacterial). Research by Delgado-Baquerizo et al. [59] also demonstrated that ecological networks and biodiversity play important roles in maintaining multiple functions in global natural ecosystems. Network complexity is closely linked to stable ecosystem functioning [82]. The interaction between plants and microorganisms can be analyzed by constructing an ecological network to investigate the relationship between network complexity and MF. For the network complexity of co-occurrence networks (Co-occurrence networks), the average degree can be used to represent it [83]. Whether it is the ecological network between plants and microorganisms or the Co-occurrence networks within fungi or bacteria, most studies focus on the “complexity” of the network (such as the number of nodes, edges, or links) [42], or some studies have used network connectivity to quantify ecological networks or Co-occurrence networks [84]. Wagg et al. [85] observed that when the same abiotic and biotic factors are considered, there is always a significant positive correlation between network complexity and soil ecosystem multifunctionality, indicating that more complex microbial networks contribute more substantially to soil ecosystem multifunctionality.

The interaction between aboveground and belowground communities is closely related to plant regeneration and nutrient transformation. When simultaneously considering the effects of intra-domain and inter-domain ecological networks on soil ecosystem multifunctionality, it was found that both microbial network complexity and inter-domain ecological network complexity significantly influence soil ecosystem multifunctionality. This may be related to the high sensitivity of soil bacteria to changes in soil nutrients [42]. Soil bacterial communities play a crucial role in biogeochemical cycles [54]. Key nitrogen transformation processes such as soil nitrogen fixation, nitrification, denitrification, and ammonification are predominantly mediated by soil bacteria [54]. The composition and activity of bacteria primarily depend on soil physical and nutrient conditions, making them more sensitive to changes in these factors. Additionally, soil bacteria are involved in multiple nutrient cycles (e.g., carbon cycling, biological nitrogen fixation, and denitrification; [41]). Therefore, changes in forest land use intensity significantly alter soil nutrients and physicochemical properties, thereby profoundly affecting soil bacteria, particularly their interactions.

## 4. Effects of Environmental Changes on Soil Ecosystem Multifunctionality

Biotic and abiotic factors can influence ecosystem functions either directly or indirectly [13,86]. For example, abiotic factors can directly affect ecosystem functions by enhancing the activities and interactions among their consumers, detritivores, decomposers, and microorganisms [55], and indirectly influence these functions by altering community composition [74]. Abiotic factors can also shift the balance among pathogenic, saprophytic, and mutualistic taxa that regulate nutrient availability for plants [87]. The stability of typical ecosystems on the Qinghai–Tibet Plateau is primarily determined by environmental factors, which can directly influence ecosystem stability rather than acting solely through biodiversity pathways [88]. While many local-scale studies suggest that environmental conditions affect ecosystem stability indirectly via biological mechanisms, recent evidence indicates that certain abiotic factors—such as water availability—exert a direct impact on ecosystem stability. Alterations in abiotic conditions can lead to substantial changes in the stability of various ecosystems, including alpine meadows, deserts, shrublands, and grasslands, with observed stability fluctuations of 43%, 40%, 52%, and 36%, respectively [89]. Moreover, Li et al.’s [42] study demonstrates that during the dry season, soil water content emerges as the most critical determinant of soil ecosystem multifunctionality, boasting the highest importance value among all assessed factors.

Environmental changes can significantly influence ecosystem functions and multifunctionality by altering ecosystem biomass and diversity [90]. Soil microbial diversity and biomass also exhibit seasonal variations, impacting both individual ecosystem functions [91] and soil ecosystem multifunctionality [92]. Climate can modulate the relationships between soil microbial or plant diversity and soil ecosystem multifunctionality at regional scales [30]. Temperature and moisture are likely key factors driving the seasonal dynamics of forest soil microbial communities [93]. Temperature and precipitation can alter ecosystem processes, including nutrient cycling, primary productivity, and biodiversity [94]. Both temperature and moisture are closely linked to biochemical processes and microbial metabolic rates [94], directly shaping microbial communities and ecological networks by regulating microbial metabolic activity, assembly, and evolution, thereby influencing integrated metabolic pathways supported by interactions among biological groups [95]. Precipitation can affect soil ecosystem multifunctionality through plant species richness, soil pH, soil water content, and soil biodiversity [30]. As a primary limiting factor for plant growth, water availability represents the most significant constraint for the restoration of arid and degraded ecosystems [96]. Research has shown that soil water content and temperature significantly influence plant and microbial community composition and diversity, as well as the complexity of inter-domain and intra-domain ecological networks [19]. Differences in microbial network complexity and soil ecosystem multifunctionality between dry and wet seasons may be linked to water stress. Therefore, soil water content plays a crucial role in microbial community structure and ecosystem functions. The findings of de Vries et al. [97] suggest that vegetation changes may alter microbial community composition by modifying soil water content, which further impacts ecosystem functions and multifunctionality. Soil water content and air temperature can directly affect soil ecosystem multifunctionality and indirectly influence it through the mediation of soil microbial diversity and network complexity [42].

## 5. Conclusions

**Figure 1:**
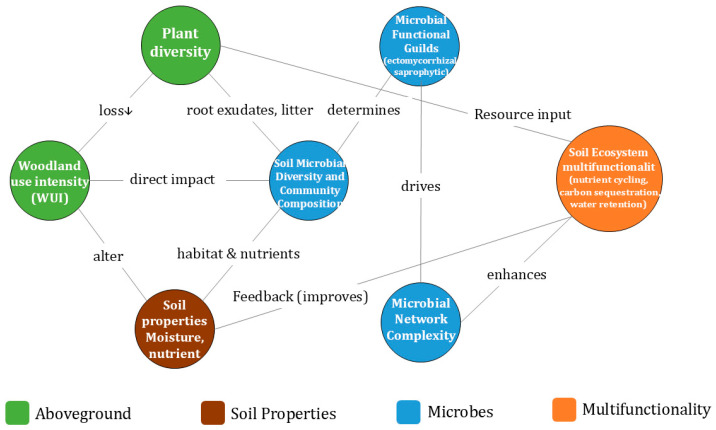
A generalized diagram of how woodland use affects the multifunctionality of soil ecosystem.

Woodland use intensity profoundly influences the diversity and composition of soil microbial communities. Increased WUI is a pivotal driver of changes in forest soil microbial communities. Studies indicate that heightened WUI typically reduces forest plant diversity, directly affecting root exudates and litter inputs and consequently altering soil microbial habitats. Specifically, rising utilization intensity often disrupts the original forest-fungal symbiotic relationships (e.g., ectomycorrhizal fungi), leading to a decline in the relative abundance of ectomycorrhizal fungi while increasing that of saprotrophic fungi. This shift in community structure modifies carbon and nitrogen cycling pathways (e.g., saprotrophic fungi dominance may accelerate organic matter mineralization) and potentially impacts long-term soil carbon storage. Additionally, soil bacterial communities (e.g., Acidobacteria and Proteobacteria) are regulated by changes in soil phosphorus, nitrogen content, and pH, further influencing nitrogen transformation processes. Overall, WUI reshapes soil microbial diversity and functional gene pools through alterations in soil physicochemical properties and plant-derived resource inputs (Figure 1).

Woodland use intensity affects soil ecosystem multifunctionality through biotic–abiotic interactions. WUI influences soil ecosystem multifunctionality (Ecosystem Multifunctionality) through complex biotic-abiotic interaction networks. Specific mechanisms include: (1) Biodiversity Regulation: Reduced plant diversity weakens the complementarity effect, leading to declines in functions such as soil water retention and nutrient cycling. (2) Ecological Network Complexity: The complexity of microbial internal and cross-domain (fungi-bacteria) ecological networks is crucial for maintaining soil multifunctionality; higher network complexity typically corresponds to more stable and efficient ecological functions. (3) Mediating Role of Environmental Factors: Abiotic factors like temperature, precipitation, and soil moisture regulate microbial metabolic rates and community structure, indirectly influencing multifunctionality (e.g., water stress during dry seasons may reduce network complexity). Therefore, WUI’s impact on soil ecosystems is multidimensional, involving a full-chain effect from microbial functional shifts to macroscopic ecosystem services.

In summary, woodland use can affect ecosystem multifunctionality not only through the relationship between aboveground biodiversity and ecosystem functions but also indirectly by leveraging belowground soil microbial diversity, as well as the interactions between plants and microorganisms. We recommend that future studies on ecosystem multifunctionality should strengthen the consideration of environmental factors. Future research could focus on how the soil ecosystem multifunctionality of typical forests responds to global changes across different scales. This includes enhancing the study of the comprehensive effects and underlying mechanisms of biodiversity and multidimensional environmental factors on soil ecosystem multifunctionality. It is also recommended that relevant research should promptly incorporate new concepts and standardize the calculation methods for soil ecosystem multifunctionality.

## Data Availability

No new data were created or analyzed in this study. Data sharing is not applicable to this article.
